# Fault Restoration of Six-Axis Force/Torque Sensor Based on Optimized Back Propagation Networks

**DOI:** 10.3390/s22176691

**Published:** 2022-09-04

**Authors:** Xuhao Li, Lifu Gao, Xiaohui Li, Huibin Cao, Yuxiang Sun

**Affiliations:** 1 Institutes of Physical Science and Information Technology, Anhui University, Hefei 230031, China; 2 Hefei Institutes of Physical Science, Chinese Academy of Sciences, Hefei 230031, China; 3 Department of Science Island, University of Science and Technology of China, Hefei 230026, China; 4 Beijing Institute of Control Engineering, Beijing 100080, China

**Keywords:** force/torque sensor, back propagation neural network, fault restoration, coupling, particle swarm optimization

## Abstract

Six-axis force/torque sensors are widely installed in manipulators to help researchers achieve closed-loop control. When manipulators work in comic space and deep sea, the adverse ambient environment will cause various degrees of damage to F/T sensors. If the disability of one or two dimensions is restored by self-restoration methods, the robustness and practicality of F/T sensors can be considerably enhanced. The coupling effect is an important characteristic of multi-axis F/T sensors, which implies that all dimensions of F/T sensors will influence each other. We can use this phenomenon to speculate the broken dimension by other regular dimensions. Back propagation neural network (BPNN) is a classical feedforward neural network, which consists of several layers and adopts the back-propagation algorithm to train networks. Hyperparameters of BPNN cannot be updated by training, but they impact the network performance directly. Hence, the particle swarm optimization (PSO) algorithm is adopted to tune the hyperparameters of BPNN. In this work, each dimension of a six-axis F/T sensor is regarded as an element in the input vector, and the relationships among six dimensions can be obtained using optimized BPNN. The average MSE of restoring one dimension and two dimensions over the testing data is $1.1693\times 10^{-5}$ and $3.4205\times 10^{-5}$, respectively. Furthermore, the average quote error of one restored dimension and two restored dimensions are $8.800\;{\times \;10}^{-3}$ and $8.200\;{\times \;10}^{-3}$, respectively. The analysis of experimental results illustrates that the proposed fault restoration method based on PSO-BPNN is viable and practical. The F/T sensor restored using the proposed method can reach the original measurement precision.

## 1. Introduction

Multi-axis F/T sensors play an important role in high-end manufacturing fields. For example, F/T sensors can provide feedback for researchers to achieve fine grab in deep sea and the remote control in comic space [1,2,3]. However, high pressure under deep sea and high vacuum, high-low temperatures in comic space and other adverse factors will cause damage to F/T sensors [4]. It can be helpful and economical if the damaged F/T sensors can be restored rather than replaced when the damage is not fatal. Therefore, fault restoration is meaningful and necessary for multi-axis F/T sensors.

A six-axis strain gauge sensor is taken as an example of multi-axis F/T sensors in this paper, and the proposed method can be applied to other types of multi-axis F/T sensors with minor modifications. An elastic body and several strain gauges constitute the six-axis F/T strain gauge sensor, and every four strain gauges (two strain gauges for semi-bridge) pasted on an elastic body compose a Wheatstone bridge. Because all strain gauges are pasted on the same elastic body, applying a load in one direction will induce deformation not only on the corresponding dimension, but also on other dimensions, which is called coupling. The coupling effect will reduce the measurement precision of six-axis F/T sensors, and scholars have proposed numerous decoupling methods to eliminate the effects of coupling. Song et al. [5] proposed a robust static decoupling algorithm for 3-axis force sensors based on ε-SVR, and Liang et al. [6] proposed a decoupling method based on parallel voltage extreme learning machine (PV-ELM) for six-axis F/M sensors. Nevertheless, if we make use of the coupling effect and find the correlations among all dimensions, it is feasible to achieve restoration of slight damages of F/T sensors.

The key to fault restoration for F/T sensors is to find out how the output of one dimension is affected by the loads from other dimensions. Mechanical analysis and finite element analysis (FEA) can reveal inherent correlations between dimensions. Niu et al. [7] analyzed the structure of a six-dimensional parallel-mechanism force sensor and proposed a new structure to minimize the coupling effect. Nevertheless, mechanical analysis and FEA are costly and demand too much prior knowledge. Machine learning has attracted much attention recently. Neural network is a major branch of machine learning, which is widely used for regression and classification in engineering, economics, and other fields [8,9].

Back propagation (BP) neural networks have simple structures and high efficiency, which makes them popular in fault detection and restoration [10,11,12,13]. BPNNs pass information through multiple hidden layers and calculate gradients of weights and biases by the back-propagation algorithm. Hyperparameters such as the counts of layers and the width of hidden layers will effect the convergence rate and performance of BPNN. Hyperparameters are normally selected by blind trails which are costly and not satisfying. Particle swarm optimization is a famous multi-objective optimization algorithm, and it is utilized for acquiring optimal solutions in math and engineering [14,15,16,17]. Lin et al. [18] proposed an adaptive dissipative particle swarm optimization (ADPSO) algorithm, which is used to solve the resource balancing optimization problem for different network-plans scales.

In this work, we proposed a fault restoration method based on the particle swarm optimization (PSO) algorithm optimizing back propagation neural networks (BPNN). After that, we conducted a coupling experiment to research the impact of coupling effects and obtain the transfer expression for the six-axis F/T sensor. Finally, some simulations are conducted to evaluate the proposed method. The rest of this paper is organized as follows: the concept and algorithm of BPNN and the PSO algorithm are briefly reviewed, then a novel fault restoration method for six-axis F/T sensors is proposed based on PSO-BPNN. Experiments including sensor calibration and model training are presented. Finally, the performance of the proposed method is discussed through the analysis of experiment results.

## 2. Methodology

### 2.1. Back Propagation Neural Networks

**Figure 1:**
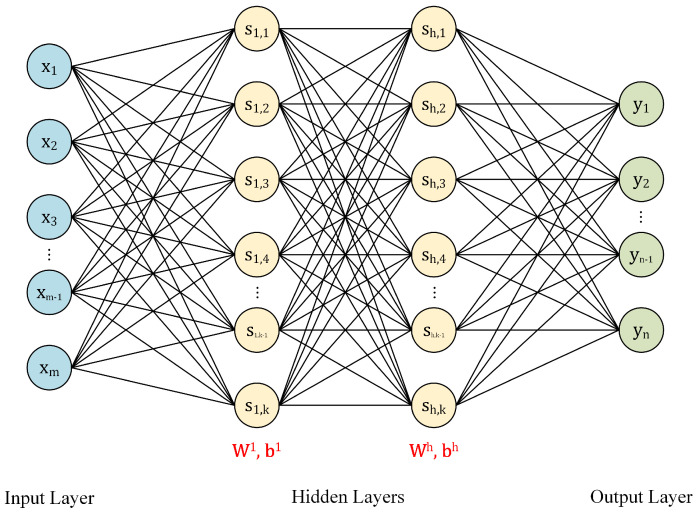
A typical structure of BPNN, W and b are the weight matrix and the bias vector, respectively.

A BP neural network consists of one input layer, one output layer and several hidden layers. Each hidden layer owns an activation function, which calculates responses of layer nodes. Assuming $X={\left\{x_{1},x_{2},\cdots ,\;x_{m}\right\}}^{T}$ is the input vector, $Y={\left\{y_{1},y_{2},\cdots ,\;y_{n}\right\}}^{T}$ is the output vector, the typical structure of BPNN is shown in Figure 1.

#### 2.1.1. Forward Propagation

The input data flows through a series of hidden layers in turn, and the output of hidden layers can be expressed as follows:

(1) $$u^{h}=f\left(W^{h}\times X+b^{h}\right),$$

where

$h=\left\{1,2,\cdots ,H\right\}$ is the index of layer,
$W^{h}\in R^{n\times l}$ and $b^{h}\in R^{l}$ are the weight matrix and the bias vector of h’th layer, respectively,
f(·) the nonlinear activate function.

The model output Y can be obtained by the same formula in (1), except that the output activation function is set to linear function:

(2) $$\hat{Y}=f\left(V\times u^{h}+b^{o}\right),$$

where $V\in R^{m\times n}$ and $b^{o}\in R^{n}$ are the weight matrix and bias vector of the output layer.

#### 2.1.2. Backward Propagation

The training principle of BPNN is to reduce the total errors in the dataset. Mean square error (MSE) is a common criterion for regression tasks, which is formulated as follows:

(3) $$loss=MSE\left(Y,\hat{Y}\right)=\frac{1}{l}\sum_{i=1}^{l}{\left(y_{i}-{\hat{y}}_{i}\right)}^{2},$$

BPNN adopts the back-propagation algorithm to train the network, which updates weights and biases along the gradient directions.

#### 2.1.3. Repetition and Termination

The progress of forward and backward propagation are repeated iteratively, and the training terminates when it reaches the maximum iteration or the target error. The maximum iteration is essential for model training, and a suitable maximum iteration should be selected for keeping balance between under-fitting and over-fitting.

### 2.2. Particle Swarm Optimization

Meta-heuristic algorithms have attracted a lot of attention in many fields, such as mathematics, cyber-security, and the Internet of Things (IoT) [19,20]. These algorithms have three attractive characteristics: simplicity, flexibility, and the ability to avoid local optima. The particle swarm optimization algorithm is a meta-heuristic optimization algorithm, and it is inspired by crowd behavior observed in insects, fishes and birds.

Assuming $u_{i}=\left(u_{i1},u_{i2},\cdots ,u_{in}\right)$, $v_{i}=\left(v_{i1},v_{i2},\cdots ,v_{im}\right)$ are the current position, and the speed of iteration *i*, respectively. $p_{i}=\left(p_{i1},p_{i2},\cdots ,p_{im}\right)$ presents the optimal position and $p_{i}^{*}=\left(p_{i1}^{*},p_{i2}^{*},\cdots ,p_{im}^{*}\right)$ is the global optimal position of each particle in iteration *i*. The basic evolution expression can be formulated as follows:

(4) $$\begin{array}{c} v_{id}\left(t+1\right)=w\times v_{id}\left(t\right)+c_{1}\times r_{1}\times \left(p_{id}\left(t\right)-x_{id}\left(t\right)\right)\\ +c_{2}\times r_{2}\times \left(p^{*}\left(t\right)-x_{id}\left(t\right)\right), \end{array}$$

(5) $$x_{id}\left(t+1\right)=x_{id}\left(t\right)+v_{id}\left(t+1\right),$$

where i={1,2,⋯,n} is the index of particle, d={1,2,⋯,D} is the dimension index; t={1,2,⋯,T} indicates the current generation and *w* is inertia factor; and $r_{1}$,$r_{2}$ are random variables in the range of [0,1].

The inertia factor *w* is negative. The bigger *w* means the better global search ability, while the smaller *w* means the better local search ability. Generally, dynamic strategies are adopted to adapt the inertia factor *w* to the searching progress, which is shown as follows:

(6) $$w\left(t\right)=\left(w_{max}-w_{min}\right)\cdot \frac{\left(T-t\right)}{T}+w_{min},$$

where $w_{max}$ and $w_{min}$ are the maximum and minimum inertia factor, respectively. *T* indicates the maximum iteration.

The pseudo code of PSO with dynamic strategy is shown in Algorithm 1.
**Algorithm 1:** Dynamic Particle Swarm Optimization**Inputs:**The count of dimensions *D*, the count of particles *N*,
the maximum iteration *T*, the boundary of inertia factor $\left[w_{min},w_{max}\right]$.**Outputs:**The optimal position of all dimensions.**Process:**
1.**for** each particle *i*2.    Initialize velocity $v_{i}$ and position $x_{i}$, set $p_{i}=x_{i}$;3.    Evaluate the fitness;4.**end for**5.$p^{*}=min\left(p{}_{i}\right)$;6.**while**t≤T7.    **for** i=1 to *N*8.      Update the velocity and position by (4) and (5);9.      Update the inertia factor by (6);10.      Evaluate the fitness of particle *i*;11.      **if** $fitness\left(x_{i}\right)<fitness\left(p_{i}\right)$12.         $p{}_{i}=x_{i}$;13.      **if** $fitness\left(p_{i}\right)<fitness\left(p^{*}\right)$14.         $p^{*}=p_{i}$;15.    **end for**16.**end while**17.**return**$p^{*}$

## 3. Fault Restoration Based on PSO-BPNN

The target for fault restoration is predicting the output of the broken dimension based on the correlations among other dimensions. Considering the complex of mutual impacts of coupling among dimensions, two schemes are assumed in this work: one arbitrary broken dimension restored by the other five dimensions and two arbitrary broken dimensions restored by the other four dimensions.

This chapter will describe how to apply the BPNN for restoring the broken dimension in the aforesaid two schemes and tuning hyperparameters of BPNN by the PSO algorithm. Lastly, the flowchart of the proposed method is presented.

### 3.1. Dataset Preparation

Assuming the input vectors are $X_{i}={\left[x_{i}^{1},x_{i}^{2},\cdots {,x}_{i}^{m}\right]}^{T}$, and the output vectors are $U_{i}={\left[u_{i}^{1},u_{i}^{2},\cdots {,u}_{i}^{n}\right]}^{T}$, where $\left(m,n\right)\in \left\{(5,1),(4,2)\right\}$. For the first scheme which restores one damaged dimension by the other five dimensions, input vectors contain voltages of five dimensions, and output vectors contain voltages of the broken dimension. For the second scheme, which restores two damaged dimensions by another four dimensions, input vectors contain voltages of four dimensions, and output vectors contain voltages of the two broken dimensions. The data is collected in coupling experiments by exerting loads orderly on dimensions. About 70% of collected data will be separated for training, while the remaining 30% for testing.

### 3.2. The BP Network

#### 3.2.1. Network Structures

The basic BP network consists of one input layer, one output layer and one hidden layer. Besides, the amount of hidden layer elements is selected by experience. In this work, the PSO algorithm is utilized to obtain the optimal counts of hidden layers and elements in each hidden layer. The counts of elements in input and output layers are decided according to Section 3.1.

#### 3.2.2. Activation Function

Activation function g(z) for all hidden layers is the hyperbolic tangent activation function, which is formulated as follows:

g(z)=tanh(z).

Additionally, the linear function is selected for the output layers, which is shown as the following equation:

g(z)=z.

#### 3.2.3. Cost Function

The BP networks use a cost function to calculate the error between predicted values and real values. Mean square error is selected to be the cost function in this work, which is suitable for regression task.

#### 3.2.4. Maximum Iteration

The BP networks will update weights and bias during training progress iteratively. Early Stopping, a type of parameter fine tuning strategy, calculates the accuracy of a model at the end of each cycle and stop training when the accuracy is no longer increasing. Hence, appropriate iterations can benefit to alleviate the under-fitting or over-fitting problems. The PSO algorithm is utilized to search for the optimal target iteration, which enhances the model performance.

### 3.3. Model Optimization by PSO

This section will illustrate how PSO is applied to optimize hyperparameters for the BPNN. As shown in Section 3.2, there are three hyperparameters to be optimized in BPNN, including the counts of hidden layers, the counts of neurons in each hidden layer, and the maximum iterations.

#### 3.3.1. Searching Space of Parameters

Considering the balance between model complexity and performance, we selected an approximate searching range for parameters, which is listed in Table 1.

#### 3.3.2. PSO Configurations

After a large number of experiments, the particle count of PSO was set to 50. The target iteration of PSO was set to 300 according to the model precision. The update strategy of inertia factor *w* is linear decreasing, as shown in (6).

**Figure 2:**
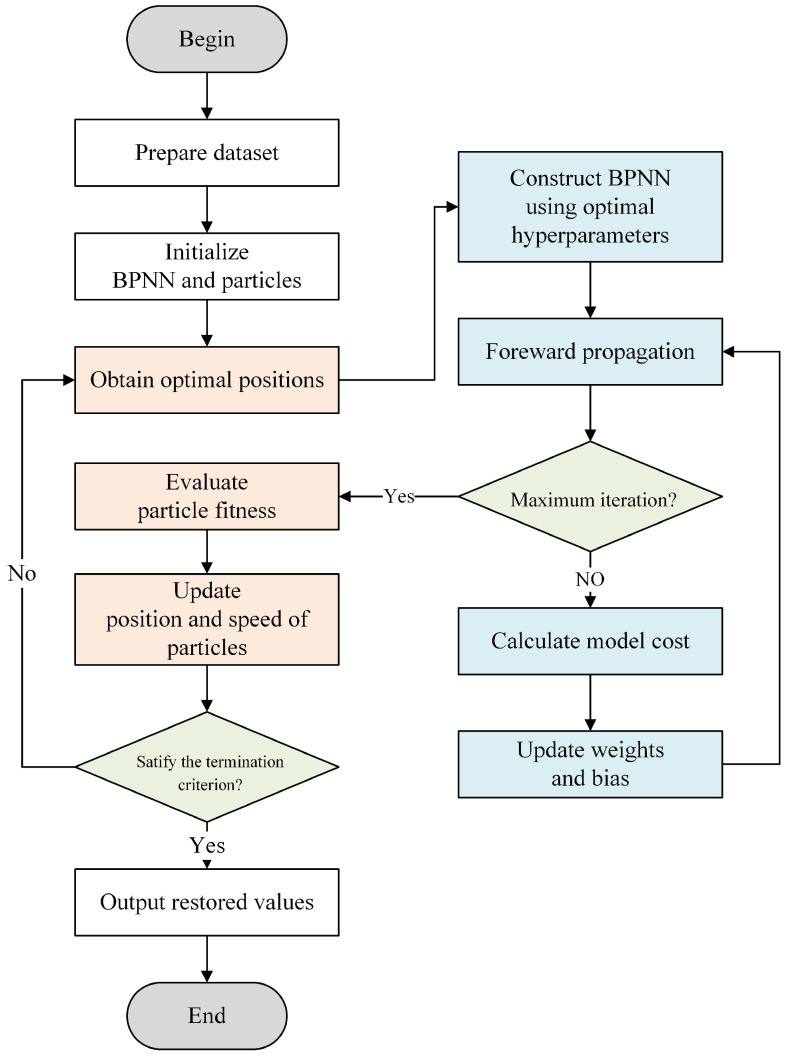
Flowchart of fault restoration for six-axis F/T sensors based on PSO-BPNN.

The implementation process of the BPNN optimized using the PSO algorithm is illustrated in Figure 2:

## 4. Experiments

**Figure 3:**
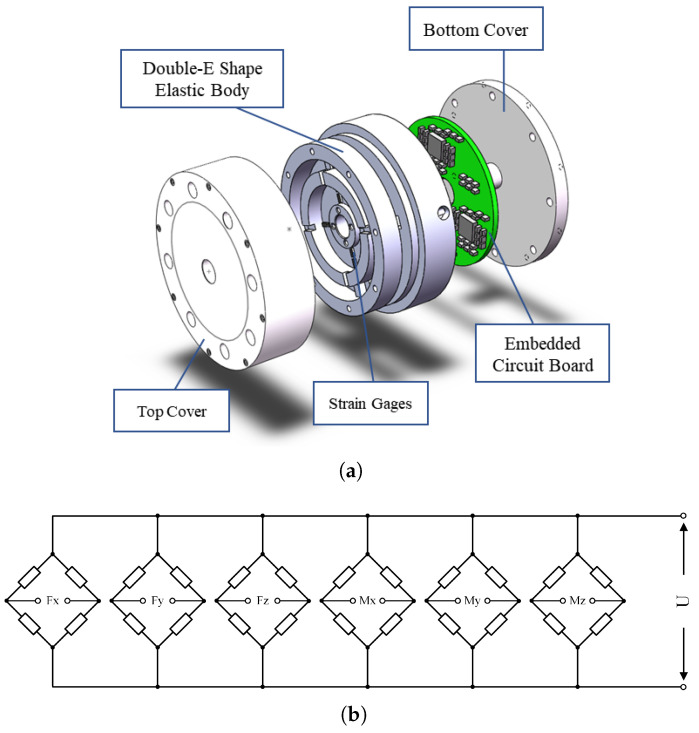
A novel six-axis F/T sensors designed by IIM, CAS. (**a**) The six-axis F/T sensor prototype. (**b**) The measurement circuit consists of six Wheatstone bridges.

A six-axis F/T sensor produced by the Institute of Intelligent Machines (IIM), Chinese Academy of Sciences (CAS), is utilized for experiments in this section. A unique double E-shape elastic body and several strain gauges constitute this six-axis F/T sensor [21]. The prototype and measurement circuit of the six-axis F/T sensor is shown in Figure 3, and the rated ranges of all dimensions are shown in Table 2. The experiment was programmed using MATLAB software and conducted on a PC which contains 3.6 GHz CPU and NVIDIA RTX 3070 GPU.

### 4.1. Coupling Experiments

A coupling experiment was conducted to study the coupling effects among dimensions on the F/T sensor, and the experimental data were gathered for sensor calibration and model training. The coupling experiment is similar to the sensor calibration, which investigates the coupling output of all dimensions while the single dimension is loaded. Assuming $U={\left[u_{1},u_{2},\cdots ,u_{6}\right]}^{T}$ represents the vector of voltage outputs of six dimensions, and L=[Fx,Fy,Fz,Mx,My,Mz] indicates the measured load vector.

The main procedures of coupling and calibrating for six-axis F/T sensors apply a series of specific loads, which increase from minimum to maximum rated ranges with a certain step. These procedures were repeated three times in this coupling experiment, and the load vector *L* and voltage vector *U* were recorded at sample points for processing. The configuration of sample points is shown in Table 3, and the temperature and humidity of environment are 25 °C and 60%, respectively.

#### 4.1.1. Calibration

Calibrating for F/T sensors aims to build the transfer expression between loads and output voltages, and it can be formulated by the following equation:

(7) L=W×U+B,

where *W* is a calibration matrix, which consists of weights between loads and output voltages, and *B* is bias vector.

Least Square (LS) algorithm is commonly utilized to calculate the calibration matrix *W* and bias vector *B*. W,B obtained from LS in this calibration experiment are shown as follows:

(8) $$W=\left(\begin{array}{cccccc} 0.870 & -0.001 & 0.004 & 0.009 & -0.710 & -0.004\\ -0.009 & 0.837 & -0.03 & 0.687 & 0.005 & -0.009\\ 0.004 & 0.003 & 0.280 & -0.003 & 0.005 & 0.003\\ -0.019 & -0.304 & 0.025 & 0.597 & 0.014 & -0.005\\ 0.250 & -0.007 & 0.005 & -0.011 & 0.638 & 0.002\\ -0.101 & -0.025 & 0.006 & -0.011 & 0.087 & 1.401 \end{array}\right),$$

(9) $$B={\left[-0.925,-75.016,-49.692,5.463,164.978,-31.988\right]}^{T}.$$

The transfer expression is obtained by substituting *W* and *B* into (7).

#### 4.1.2. Coupling

**Figure 4:**
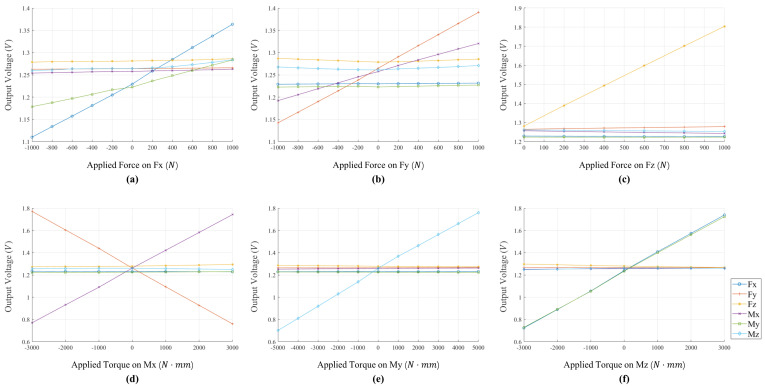
Voltage outputs of all dimensions when exerting loads on a single dimension. Not only does the loaded dimension have the output, other dimensions also have corresponding coupling outputs.

Coupling effects can be illustrated by taking the load on one dimension as an independent variable, while the output voltages of all dimensions are dependent variables. As shown in Figure 4, coupling effects are observed between some dimension pairs. For example, the output voltages of dimension My have positive correlation with the loads on dimension Fx, the output voltages of dimension Fy have a negative correlation with the loads on dimension Mx. However, the loads exerted on dimension Fz and My cause minor effects on other dimensions, which means the structures of dimension Fz and My are somewhat independent of other dimensions.

Based on the above analysis, coupling effects exist among dimensions of the six-axis F/T sensor, and we can use this phenomenon to restore one and two dimensions from damages. Besides, the effects of restoration are relative to the coupling level of dimensions.

### 4.2. Model Training

The data gathered in the coupling experiment contains the output of six dimensions. Since any dimension can be damaged in work, we assumed that one dimension was damaged in scheme 1 and restored it, then repeated this progress for all dimensions in turn. Similarly, we assumed that two dimensions were broken in scheme 2 and restored them by turns. Thus, there will be six combinations in scheme 1 and fifteen combinations in scheme 2.

To evaluate the effect of the proposed method, we take a basic BP network as a comparison, which consists of an input layer, an output layer, and two hidden layers.

### 4.3. Experiment Result and Analysis

**Figure 5:**
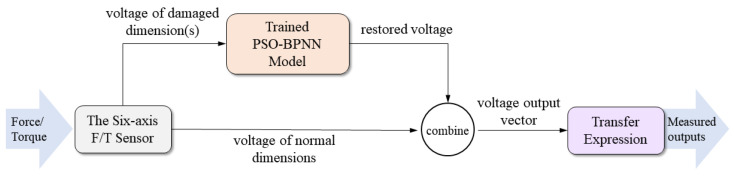
Workflow of trained PSO-BPNN in the measurement process.

**Figure 6:**
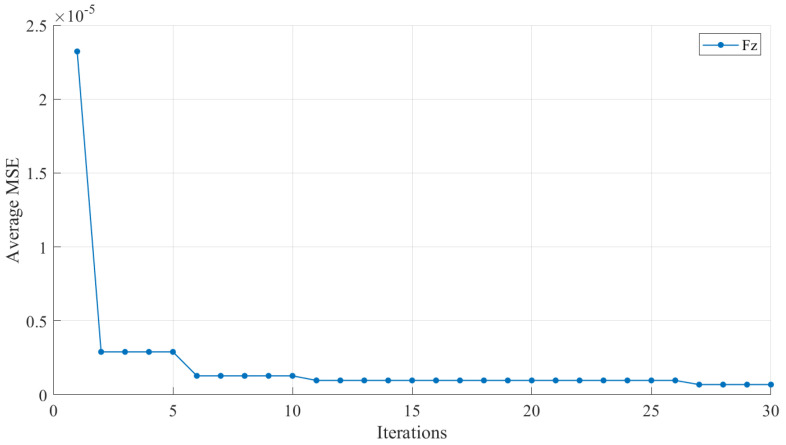
The convergence curve of training for dimension Fz.

After the restoration model is well trained, restoration experiments are carried out to evaluate the performance of the proposed method. Figure 5 shows how the trained model works in the measurement process. The testing errors indicated by the MSE of scheme 1 and scheme 2 are shown in Table 4 and Table 5, respectively. Besides, the convergence curve of training for dimension Fz is taken for a representative, which is shown in Figure 6.

Expect for the MSE, we calculated the quote errors (QE) between the measurement outputs of the normal six-axis F/T sensor and restored one. The QE divides the full scale of the factor, which can make it easier to compare the performance of dimensions whose rated ranges are different. The quote errors can be calculate using (10).

(10) $$QE\left(\hat{y},y\right)=\underset{y_{i}\in D}{average}\left(\frac{\left|{\hat{y}}_{i}-y_{i}\right|}{y_{FS}}\right),$$

where $\hat{y}$ and y indicate the restored measurement vector and original measurement vector, respectively. *D* is the training set or testing set, and $y_{FS}$ is the measurement range of the corresponding dimension.

The quote errors of scheme 1 and scheme 2 in the training and testing sets are shown in Table 6 and Table 7, respectively. The measurement ranges are listed in Table 2.

As the experiment results show in Table 4 and Table 5, the maximum and average testing errors of one dimension restoration are $5.553\times 10^{-5}$ and $1.1693\times 10^{-5}$. The maximum and average testing MSEs of two dimensions restoration are $1.218\times 10^{-4}$ and $3.4205\times 10^{-5}$. The testing MSEs show that the PSO-BPNN can accurately represent the correlations between dimensions in most conditions, and it is possible to restore the output of the damaged dimension with the remaining dimensions.

The maximum and average quote errors of one dimension are $3.600{\;\times \;10}^{-2}$ and $8.800\;{\times \;10}^{-3}$. The maximum and average quote errors of two dimensions are $2.42{\;\times \;10}^{-2}$ and $8.200{\;\times \;10}^{-3}$. The quote errors in testing show that the measurement output of the restored dimensions can reach the original precision class, which means the proposed restoration method is feasible and satisfactory.

In addition, the average fitness of BPNN during training Fz is taken to illustrate the convergence characteristic of PSO, and the convergence curve is shown in Figure 6. As can be seen from Figure 6, the fitness continued to decrease before reaching the target iterations, which means that the PSO algorithm has good global search ability and is not easy to trap in local optima.

## 5. Conclusions

Multi-axis force/torque sensors are commonly deployed in robotics and industry. Due to the bad ambient conditions, multi-axis F/T sensors will be occasionally damaged, which is fatal to the control system. If one or two dimensions get damaged, it is costly and troublesome to replace the entire F/T sensor. Hence, fault restoration is meaningful and necessary for multi-axis F/T sensors.

Back propagation neural networks are popular for their simpleness and good performance. In this work, we adopted BPNN to represent the correlation between dimensions and build restoration models for damaged dimensions. Hyperparameters are essential for BPNN, and searching for good hyperparameters is low effective. The PSO algorithm is utilized to determine the optimal structure for BPNN in the proposed model. Moreover, a coupling experiment was conducted to assess the coupling effects of six-axis F/T sensors and provide data for model training.

The results of fault restoration experiments illustrated that the PSO-BPNN is feasible and suitable for fault restoration, and the PSO algorithm can provide optimal hyperparameters to improve the performance of BPNN. Multi-axis F/T sensors, which embed the fault restoration method, are more reliable and robust when working in the bad environment.

The proposed method performs well in fault restoration, but the model does not take into account the indirect coupling effect between dimensions. In future research, we will explore the inner mechanism of coupling effects and detect damage to multi-axis F/T sensors. 

## Figures and Tables

**Table 1:** Searching ranges of hyperparameters.

Parameters	Ranges
counts of hidden layer	[1, 5]
neuron counts in each hidden layer	[5, 50]
maximum iteration	[500, 1000]

**Table 2:** Rated range of each dimension.

Dimensions	Ranges	Units
Fx	−1000to1000	N
Fy	−1000to1000	N
Fz	−1000to1000	N
Mx	−30to30	N·m
My	−30to30	N·m
Mz	−30to30	N·m

**Table 3:** The calibration experiment configuration.

Dimensions	Load Points	Units
Fx	0, ±200, ±400, ±600, ±800, ±1000	N
Fy	0, ±200, ±400, ±600, ±800, ±1000	N
Fz	0, ±200, ±400, ±600, ±800, ±1000	N
Mx	0, ±10, ±20, ±30	N·m
My	0, ±10, ±20, ±30	N·m
Mz	0, ±10, ±20, ±30, ±40, ±50	N·m

**Table 4:** Testing error of restoring one dimension.

Dimensions	PSO-BPNN	Std-BPNN
Fx	$1.066{\;\times \;10}^{-5}$	$5.518{\;\times \;10}^{-5}$
Fy	$6.888{\;\times \;10}^{-7}$	$5.711{\;\times \;10}^{-5}$
Fz	$1.914{\;\times \;10}^{-6}$	$1.193{\;\times \;10}^{-6}$
Mx	$4.147{\;\times \;10}^{-7}$	$8.711{\;\times \;10}^{-7}$
My	$7.432{\;\times \;10}^{-7}$	$8.351{\;\times \;10}^{-7}$
Mz	$5.553{\;\times \;10}^{-5}$	$9.361{\;\times \;10}^{-5}$

**Table 5:** Testing error of restoring two dimensions.

Dimensions	PSO-BPNN	Std-BPNN
Fx,Fy	$2.778\;\times \;10^{-5}$	$2.428\;\times \;10^{-5}$
Fx,Fz	$3.518\;\times \;10^{-7}$	$2.701\;\times \;10^{-6}$
Fx,Mx	$4.200\;\times \;10^{-6}$	$2.153\;\times \;10^{-6}$
Fx,My	$2.861\;\times \;10^{-5}$	$1.353\;\times \;10^{-4}$
Fx,Mz	$4.887\;\times \;10^{-5}$	$3.928\;\times \;10^{-5}$
Fy,Fz	$5.857\;\times \;10^{-7}$	$5.812\;\times \;10^{-6}$
Fy,Mx	$1.218\;\times \;10^{-4}$	$2.218\;\times \;10^{-4}$
Fy,My	$1.831\;\times \;10^{-5}$	$9.515\;\times \;10^{-6}$
Fy,Mz	$5.245\;\times \;10^{-5}$	$5.429\;\times \;10^{-5}$
Fz,Mx	$2.723\;\times \;10^{-5}$	$1.500\;\times \;10^{-4}$
Fz,My	$2.203\;\times \;10^{-6}$	$2.885\;\times \;10^{-6}$
Fz,Mz	$1.187\;\times \;10^{-4}$	$1.287\;\times \;10^{-4}$
Mx,My	$2.194\;\times \;10^{-6}$	$1.351\;\times \;10^{-5}$
Mx,Mz	$9.272\;\times \;10^{-6}$	$2.054\;\times \;10^{-5}$
My,Mz	$5.059\;\times \;10^{-5}$	$6.253\;\times \;10^{-5}$

**Table 6:** Quote errors of restoring one dimension.

Dimensions	Training Set	Testing Set
Fx	$7.524{\;\times \;10}^{-5}$	$8.192{\;\times \;10}^{-5}$
Fy	$5.419{\;\times \;10}^{-4}$	$5.863{\;\times \;10}^{-4}$
Fz	$1.193{\;\times \;10}^{-4}$	$1.454{\;\times \;10}^{-4}$
Mx	$3.900{\;\times \;10}^{-3}$	$7.000{\;\times \;10}^{-3}$
My	$7.100{\;\times \;10}^{-3}$	$8.700{\;\times \;10}^{-3}$
Mz	$4.020{\;\times \;10}^{-2}$	$3.600{\;\times \;10}^{-2}$

**Table 7:** Quote errors of restoring two dimensions.

Dimensions	Training Set	Testing Set
Fx,Fy	$2.254\;\times \;10^{-4}$	$2.885\;\times \;10^{-4}$
Fx,Fz	$1.690\;\times \;10^{-4}$	$1.993\;\times \;10^{-4}$
Fx,Mx	$3.700\;\times \;10^{-3}$	$6.300\;\times \;10^{-3}$
Fx,My	$6.300\;\times \;10^{-3}$	$7.800\;\times \;10^{-3}$
Fx,Mz	$1.690\;\times \;10^{-2}$	$1.830\;\times \;10^{-2}$
Fy,Fz	$1.498\;\times \;10^{-4}$	$2.054\;\times \;10^{-4}$
Fy,Mx	$4.400\;\times \;10^{-3}$	$1.890\;\times \;10^{-2}$
Fy,My	$2.500\;\times \;10^{-3}$	$3.300\;\times \;10^{-3}$
Fy,Mz	$1.800\;\times \;10^{-2}$	$1.890\;\times \;10^{-2}$
Fz,Mx	$4.300\;\times \;10^{-3}$	$5.900\;\times \;10^{-3}$
Fz,My	$2.300\;\times \;10^{-3}$	$2.600\;\times \;10^{-3}$
Fz,Mz	$1.560\;\times \;10^{-2}$	$2.150\;\times \;10^{-2}$
Mx,My	$6.100\;\times \;10^{-3}$	$8.000\;\times \;10^{-3}$
Mx,Mz	$2.420\;\times \;10^{-2}$	$2.880\;\times \;10^{-2}$
My,Mz	$1.760\;\times \;10^{-2}$	$1.800\;\times \;10^{-2}$

## Data Availability

Not applicable.
